# Predicting the impact of CPAP on brain health: A study using the sleep EEG‐derived brain age index

**DOI:** 10.1002/acn3.52032

**Published:** 2024-02-23

**Authors:** Soonhyun Yook, Hea Ree Park, Eun Yeon Joo, Hosung Kim

**Affiliations:** ^1^ USC Stevens Neuroimaging and Informatics Institute, Keck School of Medicine of USC University of Southern California Los Angeles California 90033 USA; ^2^ Department of Neurology Inje University College of Medicine, Ilsan Paik Hospital Goyang 10380 Korea; ^3^ Department of Neurology, Neuroscience Center, Samsung Medical Center, Samsung Biomedical Research Institute, School of Medicine Sungkyunkwan University Seoul 06351 Korea

## Abstract

**Objective:**

This longitudinal study investigated potential positive impact of CPAP treatment on brain health in individuals with obstructive sleep Apnea (OSA). To allow this, we aimed to employ sleep electroencephalogram (EEG)‐derived brain age index (BAI) to quantify CPAP's impact on brain health and identify individually varying CPAP effects on brain aging using machine learning approaches.

**Methods:**

We retrospectively analyzed CPAP‐treated (*n* = 98) and untreated OSA patients (*n* = 88) with a minimum 12‐month follow‐up of polysomnography. BAI was calculated by subtracting chronological age from the predicted brain age. To investigate BAI changes before and after CPAP treatment, we compared annual ΔBAI between CPAP‐treated and untreated OSA patients. To identify individually varying CPAP effectiveness and factors influencing CPAP effectiveness, machine learning approaches were employed to predict which patient displayed positive outcomes (negative annual ΔBAI) based on their baseline clinical features.

**Results:**

CPAP‐treated group showed lower annual ΔBAI than untreated (−0.6 ± 2.7 vs. 0.3 ± 2.6 years, *p* < 0.05). This BAI reduction with CPAP was reproduced independently in the Apnea, Bariatric surgery, and CPAP study cohort. Patients with more severe OSA at baseline displayed more positive annual ΔBAI (=accelerated brain aging) when untreated and displayed more negative annual ΔBAI (=decelerated brain aging) when CPAP‐treated. Machine learning models achieved high accuracy (up to 86%) in predicting CPAP outcomes.

Interpretation.

CPAP treatment can alleviate brain aging in OSA, especially in severe cases. Sleep EEG‐derived BAI has potential to assess CPAP's impact on brain health. The study provides insights into CPAP's effects and underscores BAI‐based predictive modeling's utility in OSA management.

## Introduction

Obstructive sleep apnea (OSA) is a prevalent sleep disorder characterized by recurring interruptions in breathing during sleep due to partial or complete airway obstruction. These disruptions, known as apneas and hypopneas, lead to intermittent hypoxemia, hypercapnia, microarousals, and fragmented sleep patterns.^1^, ^2^ The cumulative impact of these events contributes to oxidative stress, systemic inflammation, and potentially adverse effects on brain structure and function in individuals with OSA.^3^, ^4^ Numerous studies have shown that OSA is associated with significant alterations in white matter connectivity and integrity, as well as reductions in gray matter volume.^5^, ^6^, ^7^ Additionally, cognitive deficits, particularly in memory, executive function, and attention, are often evident in individuals with OSA.^8^, ^9^ The alterations in brain structure and function in individuals with OSA likely lead to accelerated brain aging.

Continuous positive airway pressure (CPAP) is the primary treatment for OSA.^10^ While CPAP has demonstrated efficacy in reducing cardiovascular risk factors and alleviating daytime symptoms, its effects on the central nervous system remain a subject of ongoing investigation. Neuroimaging research has examined the impact of CPAP therapy on the brain structure and function of OSA patients. While some studies have observed enhancements in brain volume, cerebral blood flow, and functional connectivity after CPAP treatment,^11^, ^12^, ^13^, ^14^, ^15^ others have presented contrasting results, indicating no notable changes or even potential adverse effects following the therapy.^16^, ^17^ Cognitive outcomes from CPAP treatment vary, with some studies reporting improvements in executive functions and memory,^18^, ^19^, ^20^ while the multi‐center randomized, double‐blinded trial failed to demonstrate significant improvement of neurocognitive functions.^21^

These inconsistent findings may be partly due to various factors such as study populations, treatment adherence, and duration. They also indicate existing biomarkers may not be sufficiently sensitive to assess CPAP effectiveness on brain health in OSA. Therefore, there is an urgent need to develop objective biomarkers to accurately measure brain health changes and optimize CPAP treatment for OSA patients. Currently, the field of sleep medicine relies primarily on the apnea–hypopnea index (AHI) to diagnose OSA and determine the need for CPAP therapy. However, AHI only counts the number of obstructive episodes and does not capture the neurophysiological changes related to hypoxic burden (HB), microarousals, or sleep fragmentation caused by OSA.^22^ As a result, AHI may not be a reliable indicator for assessing the impact of CPAP treatment on brain health in OSA patients.

Recent research, including ours, has effectively identified changes in neuroelectrophysiological activities during sleep across aging. Using machine learning, these characteristics are translated into a brain age index (BAI), which is the difference between predicted brain age from sleep EEG and chronological age. This metric has been linked to risks of neurodegenerative and psychiatric diseases, cognitive decline, and mortality.^23^, ^24^, ^25^ Our team has shown that BAI can indicate brain health in both healthy individuals and those with sleep disorders.^26^ Specifically, we found that OSA patients have a higher BAI than healthy sleepers due to reduced EEG power, especially in slow and delta waves. These findings suggest that alterations in BAI could serve as an objective measure of the detrimental effects of OSA and their possible reversal after CPAP treatment.

In this study, we aimed to investigate the effect of CPAP treatment on brain health in OSA patients by analyzing changes in BAI before and after treatment. We compared the annual changes in BAI between individuals who receive CPAP treatment and those who do not. To enhance the clinical applicability of the BAI, we aimed to apply a machine learning approach to predict which OSA patients are most likely to experience a decrease in BAI from CPAP treatment. Further, by analyzing BAI within the treatment group and examining various baseline characteristics of patients, we aimed to identify factors that significantly influence the effectiveness of CPAP treatment.

## Methods

### Participants, PSG, and EEG acquisitions

In this retrospective study, we chose participants from 225 OSA patients who were involved in a longitudinal polysomnography (PSG) study at Samsung Medical Center (SMC) in Seoul, South Korea, from 2015 to 2019. The time interval between baseline and follow‐up PSG ranged from 0.5 to 9 years. The study divided participants into two groups: OSA patients who received to CPAP treatment and untreated patients as a reference. The CPAP‐treated group was defined as those who maintained their CPAP treatment while regularly visiting the sleep clinic throughout the study period, with a minimum mean daily usage duration of 4 h. Compliance data were collected daily throughout the course of this study. The untreated group comprised patients who initiated CPAP but discontinued within the first 2 weeks, halting treatment until their follow‐up PSG. The CPAP device utilized was the Philips DreamStation (Philips Respironics, USA).

In this study, we examined the long‐term effects of CPAP on brain health, with a minimum 12‐month gap between baseline and follow‐up assessments. Thus, all the participants who underwent CPAP had PSG without use of CPAP at both baseline and follow‐up sleep study. Participants were >18 years old with AHI >15. Prior to PSG, clinical interviews and sleep questionnaires were conducted. A comorbidity score was calculated for each subject, considering five comorbidities associated with OSA: hypertension, coronary artery disease (angina or myocardial infarction), congestive heart failure, diabetes mellitus, and cardiac arrhythmia. Participants were excluded if they exhibited any of the following: (1) history of cerebrovascular disease, (2) other neurological or psychiatric diseases, or (3) history of OSA treatment (CPAP, oral device) for more than 2 weeks. We assessed the total HB using Azarbarzin *et al*.'s approach, which we extended to calculate HB during apnea episodes (apnea‐specific HB) and hypopnea episodes (hypopnea‐specific HB) separately (details in Fig. S2).^22^ Apnea was defined as a reduction in airflow by 90% in oronasal thermal sensor and more lasting at least 10 sec, while hypopnea was defined as a reduction in airflow by 30% in nasal pressure sensor and more lasting at least 10 sec with at least 3% oxygen desaturation or arousal.^27^ In this study, patients with more than 20% of recorded SpO_2_ below 60% were excluded from hypoxic burden calculation. SpO_2_ data below 60% were replaced to the SpO_2_ value detected right before. All patient characteristics were comprehensively recorded without any missing data. SMC Institutional Review Board approved PSG data use for this retrospective analysis (IRB No.2023–07‐007). All participants provided written informed consent prior to their inclusion in the study.

For reproducibility, we incorporated data from the Apnea, Bariatric surgery, and CPAP (ABC) study,^28^, ^29^ including 13 CPAP‐treated OSA patients (6 female) aged 27 to 63 (48.7 ± 10.3), who underwent PSG at baseline and 18‐month follow‐up.

### EEG preprocessing and artifact removal

We used sleep EEG data from eight channels: frontal (F3, F4), central (C3, C4), occipital (O1, O2), and behind the ear (A1, A2). The data were sampled at 200 Hz. A1 and A2 served as reference channels, and sleep latency intervals were excluded. Data were band‐pass filtered (0–50 Hz), and artifacts related to ECG and electrooculogram (EOG) were removed via EEG LAB's AAR plugin. We checked signal amplitude for each EEG channel and interpolated amplitudes above 5 standard deviations. Approximately 3.8% of the data required interpolation. Channels with >30% artifact data were marked as “bad” channels and replaced with opposing hemispheric channels (e.g., F3 with F4, C3 with C4, and O1 with O2). Only two cases (0.5%) in the entire dataset required this replacement. We then performed z‐score normalization by standardizing the amplitude of each EEG channel relative to the mean and standard deviation of the individual's entire data. And then, preprocessed six channels EEG data were converted into a scalogram with 2000‐bins in x‐axis and 16‐frequency bands in y‐axis, using the same method in Yook *et al*.^26^

### Calculating BAI and annual ΔBAI

To predict BAI (details in S1), we utilized a sleep EEG‐based brain age prediction model. This model, with the lowest MAE of 4.8 years (as shown in Fig. S3), outperformed other EEG‐based brain age prediction models and confirmed accelerated brain aging in OSA patients compared to healthy sleepers.^26^ Input features were generated by combining sleep stage information and sleep EEG. Sleep stage was resampled to 2000‐time bins, forming a 2000 by 1 matrix. Sleep EEG data took the form of a 2000‐bin by 16‐frequency band by 6‐channel scalograms. These two types of data were concatenated, yielding input data of 2000 by 16 by 7. This input was used for the brain age prediction model, producing predicted brain age. We then calculated the BAI, which is an indicator of a subject's relative brain health status, by subtracting their chronological age from the predicted brain age.^30^, ^31^, ^32^, ^33^ More details can be found in our original study.^26^ We computed ΔBAI as the difference between follow‐up and baseline BAI. Considering the different intervals between assessments in the SMC data, we also calculated an annual ΔBAI (ΔBAI per year).

### Statistical analysis

#### Association between CPAP and BAI

We analyzed the data from SMC and ABC to explore the longitudinal association between CPAP treatment and changes in BAI, while controlling for relevant covariates, ultimately providing insights into the impact of CPAP on BAI in OSA patients. To examine whether a long‐term CPAP treatment leads to a decrease in BAI, within the CPAP‐treated group, we performed a paired t‐test to assess the difference in BAI between the baseline PSG and the follow‐up PSG for each individual. Similarly, within the untreated OSA group, we also conducted a paired t‐test to examine the difference in BAI between baseline and follow‐up. Using linear regression, we compared ΔBAI mean and annual ΔBAI between CPAP‐treated and untreated OSA groups. We accounted for combinations of potential confounders—sex, age, body mass index (BMI), AHI, and arousal index (AI)—through adjustment, ensuring a balanced comparison and addressing differences in these variables between the two groups.

#### Association between baseline characteristics and BAI change over time in untreated OSA patients

In our study of untreated OSA patients with varied BAI change patterns over time, we sought to determine if they exhibited distinct baseline clinical characteristics. Using a linear model, we examined the association between annual ΔBAI and baseline characteristics such as BAI, age, Epworth Sleepiness Scale (ESS) score, sex, total HB, apnea‐specific HB, hypopnea‐specific HB, AI, AHI, apnea index, and hypopnea index.

#### Association of BAI change over time with baseline characteristics and CPAP adherence in CPAP‐treated OSA patients

We investigated which baseline characteristics of OSA patients contribute to a reduction in BAI after CPAP treatment. Additionally, we were interested in understanding the influence of CPAP adherence on the decrease in BAI. To this end, we employed a linear model to explore the association between annual ΔBAI and baseline characteristics such as baseline BAI, age, ESS, sex, total HB, apnea‐specific HB, hypopnea‐specific HB, AI, AHI, apnea index, and hypopnea index. Furthermore, we examined the link between annual ΔBAI and CPAP adherence measures, including the percentage of days with over 4 h of CPAP usage and the average duration of CPAP use. All statistical results were adjusted for multiple comparisons through false discovery rate (FDR) control.

#### Predicting the CPAP effectiveness on brain age

We developed a CPAP effectiveness prediction model aimed at identifying OSA patients likely to benefit from CPAP treatment. Our approach involved four key steps:

1. **Patient categorization:** The CPAP‐treated cohort (*n* = 98) was divided into good and poor responders. To prevent a bias in our predictive model due to imbalances in the data used for training, we evenly divided the groups using the median value of the annual ΔBAI as a cutoff value between the two groups.
2. **Evaluation of feature importance:** We employed the neighborhood component analysis (NCA) method^34^ to assess the significance of baseline attributes such as BAI, age, sex, AI, AHI, apnea index, hypopnea index, oxygen desaturation index (ODI), total HB, apnea‐specific HB, and hypopnea‐specific HB in distinguishing between good and poor responders.
3. **Machine learning model implementation:** We utilized various algorithms like K‐Nearest Neighbors (KNN), Neural Network (NN), Decision Tree (DT), Random Forest (RF), and Support Vector Machine (SVM) to categorize OSA patients into good or poor CPAP response groups. We explored different combinations of features to achieve the best prediction accuracy, including the full feature set, top 1, 3, and 5 features as ranked by NCA, EEG‐based features (BAI and AI), features excluding EEG data, features excluding BAI, and solely BAI‐based features.
4. **Validation process:** Results were derived using a 5‐fold cross‐validation strategy (80% training, 20% testing). For the machine learning model and feature set that demonstrated the highest accuracy, we computed metrics such as AUC, sensitivity, and specificity.

## Results

### Subject characteristics

From the initial cohort of 225 OSA patients at SMC, 120 were treated and 105 were untreated. After excluding patients who did not meet the inclusion criteria outlined in the method section, 98 treated (10 female) and 88 untreated (21 female) patients were analyzed (See Fig. S1). The mean follow‐up periods were 4.7 ± 2.5 years for the CPAP group and 4.2 ± 2.4 years for the untreated group.

Table 1 outlines the baseline characteristics of the study participants. The CPAP‐treated group exhibited more severe OSA, as indicated by higher AHI and total HB, and a higher proportion of N1 sleep but lower N2 sleep than the untreated group. It is noted that this discrepancy in severity somewhat elucidates the inherent challenges in the recruitment process for clinical studies, particularly when dealing with untreated patients. Untreated patients typically consist of individuals who initially attempted CPAP treatment but struggled to sustain adherence within the initial 2 weeks. This nonadherence can stem from various reasons, including inadequate treatment effects (which could explain the lower AHI in the untreated group), discomfort associated with the CPAP equipment, experienced side effects, or difficulties in adapting to the treatment regimen.^35^ BMI, depressive mood, ESS, the comorbidity scores, and AI did not significantly differ between two groups. The CPAP‐treated group had an average CPAP usage duration of 5.9 ± 1.0 hours per day, with 77.6 ± 18.7% of days having over 4 h of CPAP usage. We included 13 OSA patients from the ABC dataset who had received CPAP treatment. These patients had an average age of 48.7 ± 10.3 years, ESS score of 10.2 ± 5.2, total sleep time (TST) of 388.7 ± 78.0 min, and AHI of 43.1 ± 31.2 at the baseline assessment. Both baseline and 18‐month follow‐up PSG were conducted without use of CPAP for this group of patients (details in Table. S2).

**Table 1:** Baseline demographics and clinical characteristics.

	CPAP‐treated (*n* = 98)	Untreated (*n* = 88)	*p‐*value
Age	53.3 (9.5)	48.8 (11.4)	0.004
Men, No. (%)	89 (90.8)	71 (80.7)	0.056
Body mass index, kg/m^2^	26.4 (2.8)	25.7 (4.2)	0.209
Beck's depression inventory	9.8 (8.7)	11.6 (8.4)	0.268
Epworth sleepiness score	10.4 (4.9)	9.7 (5.3)	0.420
Comorbidity score	1.1 (1.1)	0.8 (1.0)	0.018
Total sleep time, min	363.9 (57.0)	370.0 (58.1)	0.470
Sleep latency, min	9.0 (16.1)	10.6 (22.5)	0.587
WASO, min	64.2 (36.9)	62.3 (52.2)	0.766
Sleep efficiency, %	83.4 (8.7)	83.8 (12.6)	0.763
N1, %	29.1 (13.8)	22.1 (12.8)	<0.001*
N2, %	49.0 (12.7)	54.5 (11.4)	0.002*
N3, %	2.4 (3.6)	3.4 (4.9)	0.091
REM, %	19.6 (6.4)	20.0 (6.9)	0.693
Apnea–hypopnea index, /h	42.1 (21.7)	23.4 (16.6)	<0.001*
Arousal index, /h	37.7 (17.4)	30.1 (36.4)	0.067
Total hypoxic burden	151.2 (160.3)	62.3 (75.5)	<0.001*

* Significant after Bonferroni correction for multiple comparison (*p* < 0.05/17).

### BAI decrease after CPAP treatment

**Figure 1:**
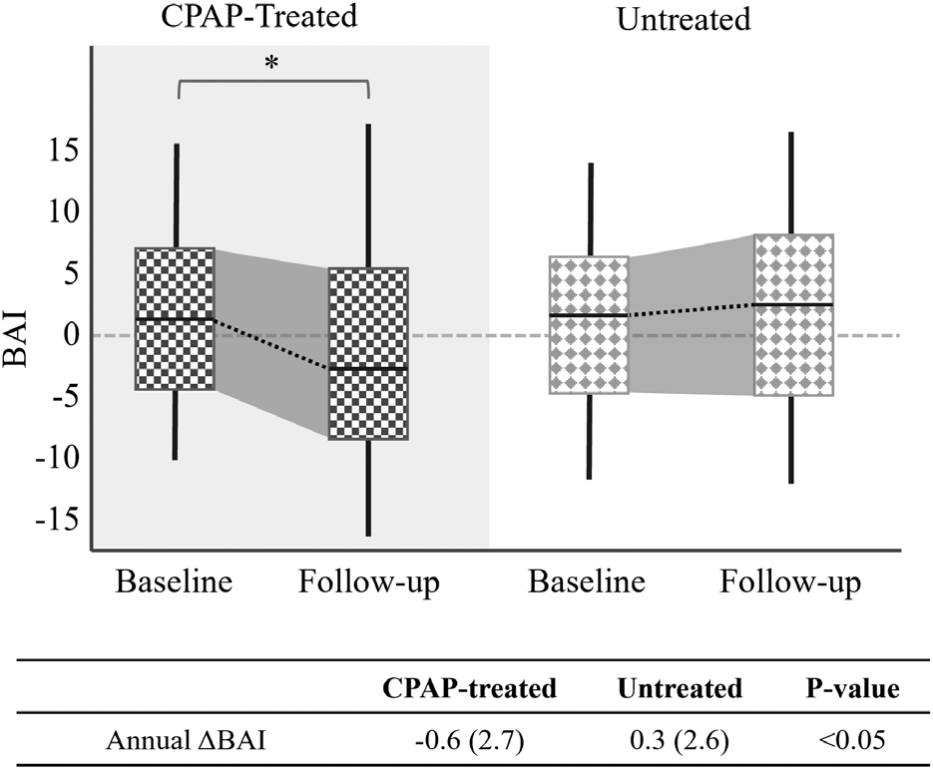
BAI comparison between CPAP‐treated and untreated OSA patients from the SMC dataset. Our results showed that the group of OSA patients undergoing a long‐term CPAP treatment (mean 4.7‐years) had a significantly lower annual ΔBAI compared to the untreated group.

In the SMC dataset, the baseline BAI exhibited no significant difference between the CPAP‐treated group (mean ± SD: 0.6 ± 6.4 years) and the untreated group (0.6 ± 6.9 years). Nevertheless, the CPAP‐treated group showed a substantial reduction in BAI from baseline to follow‐up (mean change: −1.8 ± 7.4 years, *p* < 0.05). Conversely, the untreated group presented a marginal rise in BAI (mean change: 0.6 ± 6.9 years), which was not statistically significant. The ΔBAIs (follow‐up − baseline) in the CPAP‐treated group were significantly lower than those in the untreated group (*p* < 0.05). This pattern was also consistent when accounting for the time interval (annual ΔBAI), with the CPAP‐treated group displaying a significantly lower annual ΔBAI (−0.6 years of BAI per year) compared to the untreated group (*p* < 0.05, +0.3 years). The aforementioned findings are encapsulated in Figure 1. These results were obtained after covariate adjustment for sex, age, BMI, AHI, and AI, aiming to ensure balanced data comparison between the two groups.

**Figure 2:**
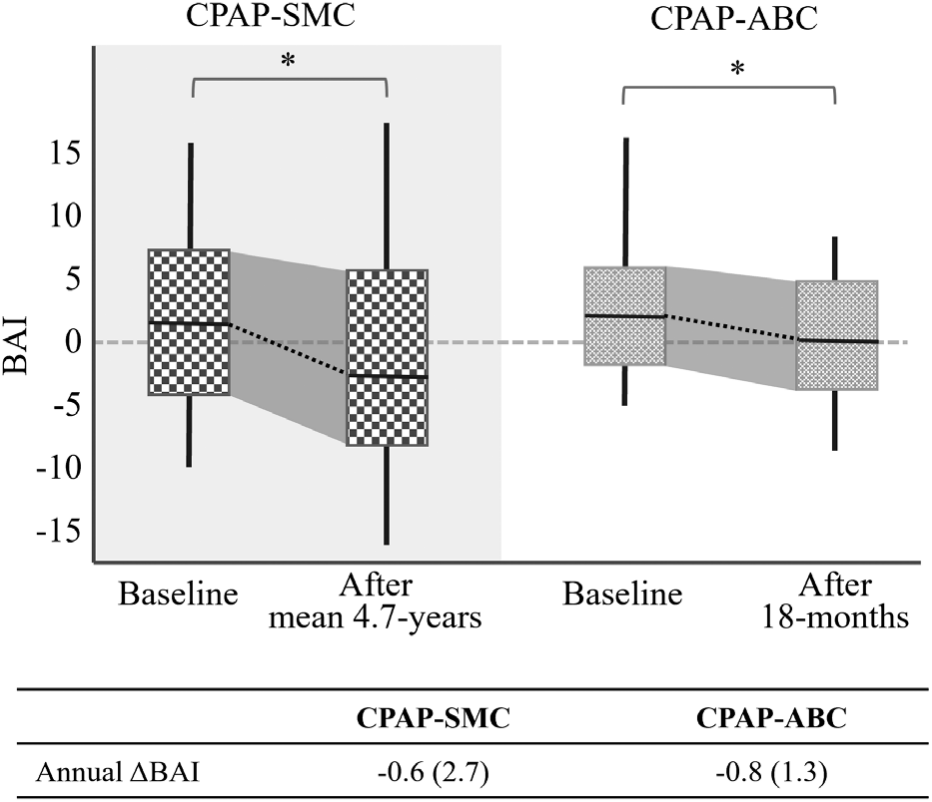
BAI change after CPAP treatment for SMC (baseline and mean 4.7‐years follow‐up) and ABC data set (baseline and 18‐months follow‐up). In both datasets, CPAP treatment led to a decrease in BAI.

In the ABC dataset, a significant decrease in BAI was observed at the 18‐month follow‐up relative to baseline, with a mean change of −1.3 ± 5.6 years (*p* < 0.05). Moreover, no significant difference in annual ΔBAI emerged between OSA patients undergoing CPAP treatment in the SMC dataset and those in the ABC dataset, even though their mean follow‐up durations differ (4.7 vs. 1.5 years, Fig. 2). Thus, the annual rate of brain age changes was not dependent on the duration of treatment.

### Baseline characteristics associated with an increase in BAI of untreated OSA patients

**Figure 3:**
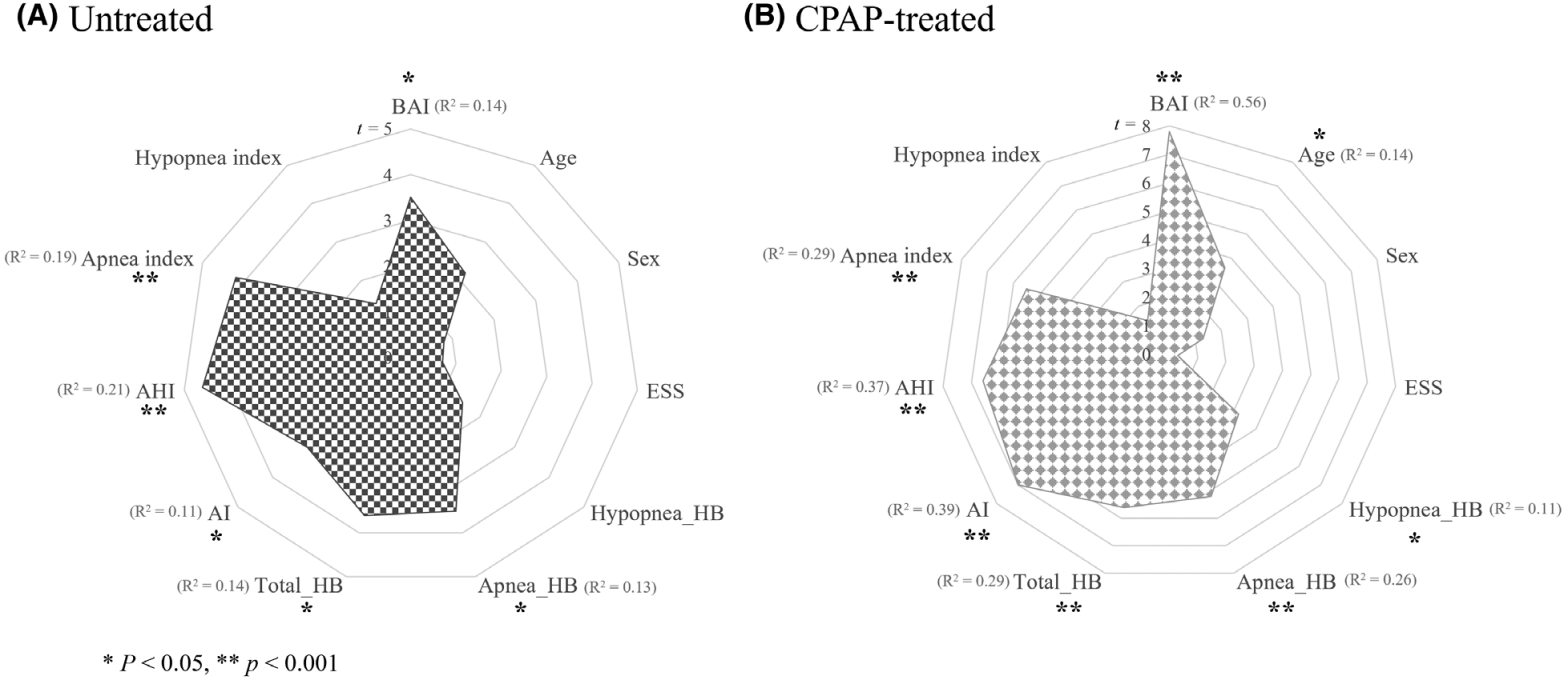
Association of baseline characteristics and adherence indices for the CPAP‐treated group with annual ΔBAI in untreated OSA patients (A) and CPAP‐treated patients (B). (A) In untreated OSA patients, high annual ΔBAI (or increased BAI) are associated with high baseline BAI, apnea‐specific HB, total HB, AI, AHI, and apnea index. (B) In the CPAP‐treated group, low annual ΔBAI (or decreased BAI) are associated with high baseline BAI, age, hypopnea‐specific HB, apnea‐specific HB, total HB, AI, AHI, and apnea index. AHI, Apnea–hypopnea index; AI, arousal index; BAI, brain age index; ESS, Epworth Sleepiness Scale; HB, hypoxic burden.

Untreated OSA patients from the SMC dataset with an increased BAI over time per year (annual ΔBAI) were significantly associated with higher baseline values in the following order: AHI (*t* = 4.6, *p* < 0.001), apnea index (*t* = 4.2, *p* < 0.001), total HB (*t* = 3.6, *p* < 0.05), apnea‐specific HB (*t* = 3.5, *p* < 0.05), BAI (*t* = 3.5, *p* < 0.05), and AI (*t* = 3.0, *p* < 0.05). Conversely, baseline age, ESS, and hypopnea‐related indices such as hypopnea‐specific HB, hypopnea index, as well as sex were not associated with BAI change (Fig. 3A).

### Baseline characteristics associated with a decrease in BAI after CPAP treatment

Our analysis showed that OSA patients with decreased BAI following long‐term CPAP use had higher baseline values in the following sequence: BAI (*t* = 7.8, *p* < 0.001), AI (*t* = 7.0, *p* < 0.001), AHI (*t* = 6.6, *p* < 0.001), total HB (*t* = 5.6, *p* < 0.001), apnea index (*t* = 5.5, *p* < 0.001;), apnea‐specific HB (*t* = 5.2, *p* < 0.001), age (*t* = 3.6, *p* < 0.05), and hypopnea‐specific HB (3.2, *p* < 0.05) In contrast, baseline ESS, hypopnea index, and sex were not associate with BAI change due to CPAP treatment (Fig. 3B). Additionally, adherence indices including the percentage of days with over 4 h of CPAP usage and the average duration of CPAP use did not associated with BAI change.

As the follow‐up PSG was performed without CPAP, no significant changes were observed in AHI or other sleep parameters, as similar as in previous studies (Details in Table S1). Furthermore, Δ values of these sleep parameters, including ΔAHI, did not show significant correlations with the ΔBAI.

### CPAP effectiveness prediction based on baseline features

**Figure 4:**
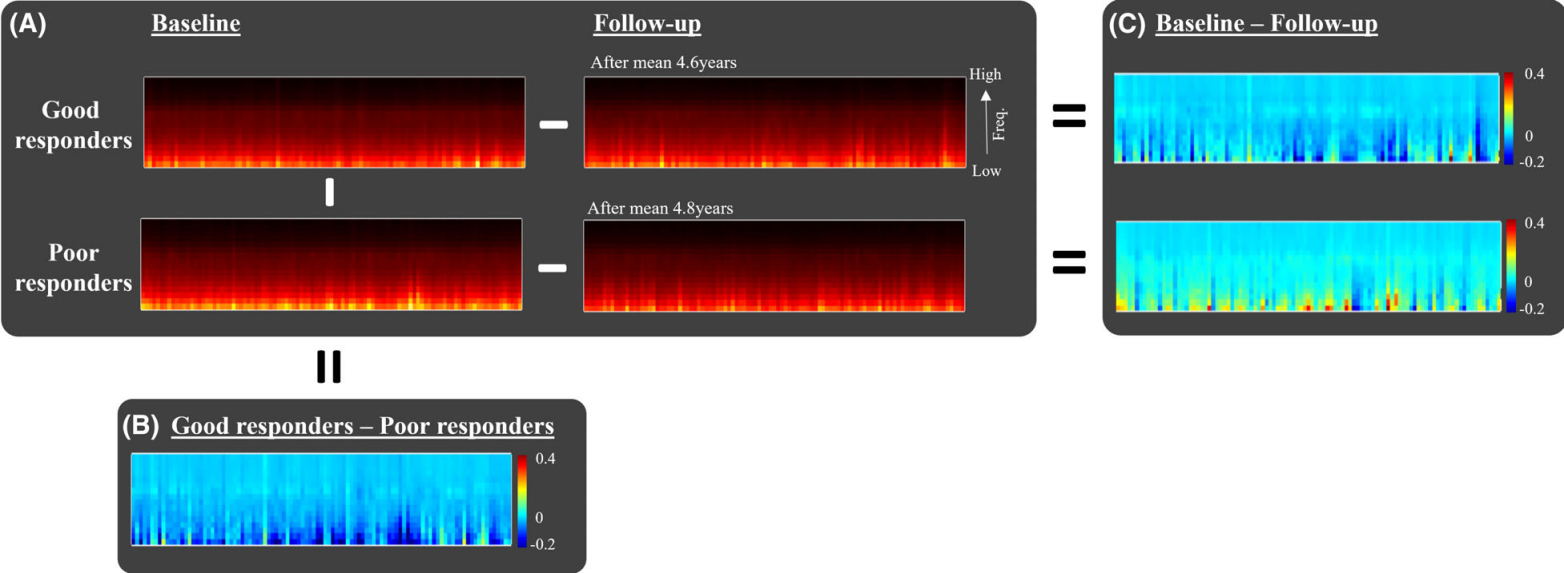
Scalogram patterns and response characteristics in good and poor responders. (A) Scalograms averaged from good responders (*n* = 49) at both baseline and follow‐up (top) and poor responders (*n* = 49) at baseline and follow‐up (bottom). (B) Subtracting poor responders' baseline scalogram from good responders' baseline scalogram unveils a pattern of negative power in lower frequency wave components. (C) Subtracting follow‐up from baseline scalogram reveals that good responders exhibit a pattern of negative power in lower frequency wave components. In contrast, poor responders display a pattern of positive power in lower frequency wave components.

To predict which OSA patients would benefit from long‐term CPAP treatment with decreased BAI, we categorized CPAP‐treated OSA patients from the SMC dataset into two groups using the median value of −0.19: good responders (annual ΔBAI ≤−0.19, *n* = 49) and poor responders (annual ΔBAI >−0.19, *n* = 49). The scalogram patterns for these two responder groups at both baseline and follow‐up are depicted in Figure 4.

**Figure 5:**
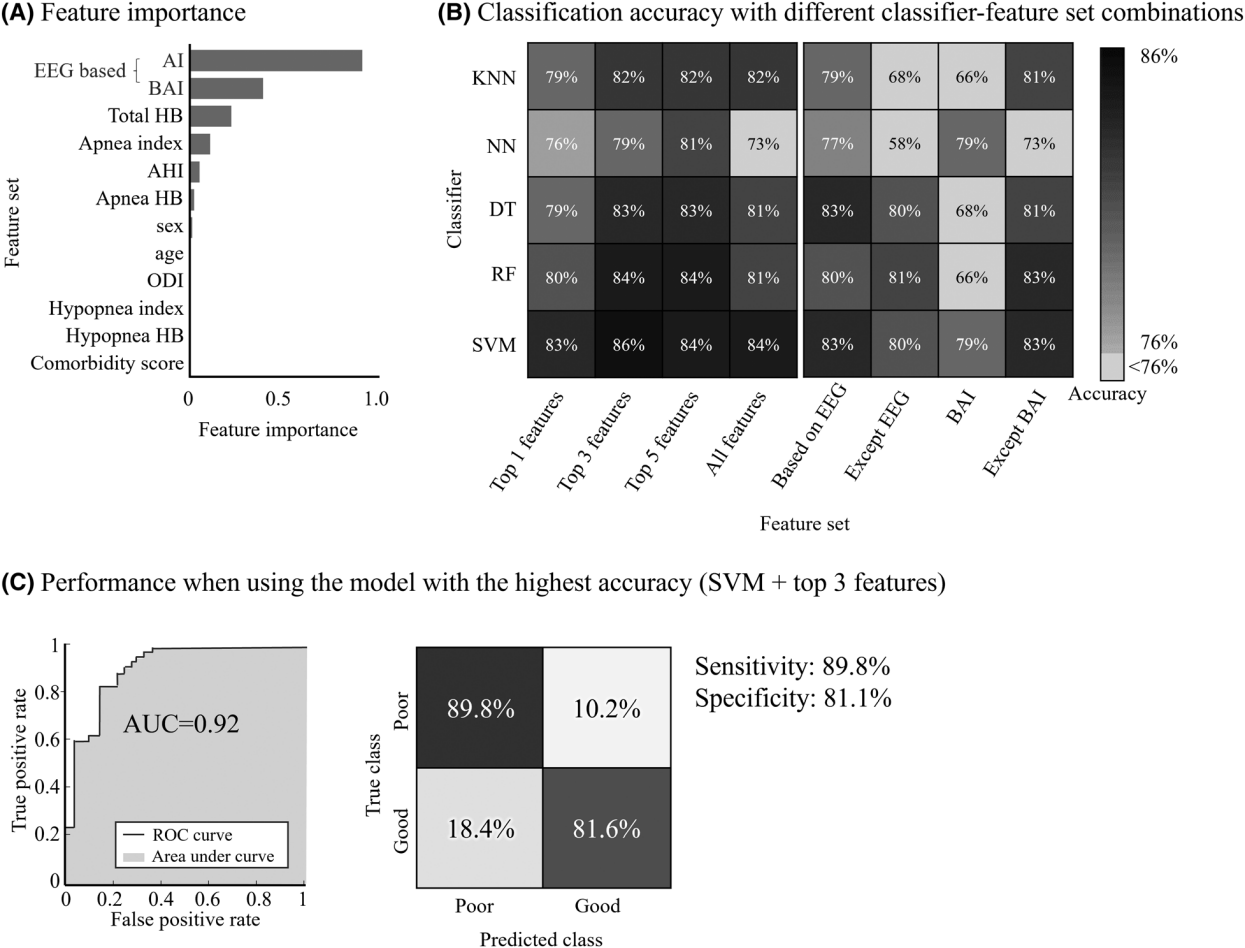
Performance of CPAP effectiveness prediction. (A) Importance of baseline features in predicting the effectiveness of CPAP using NCA. The highest importance score was observed for AI, followed by BAI, total HB, apnea index, AHI, and apnea‐specific HB. (B) Accuracy metric based on five classifiers and eight combinations of feature sets. The highest performance accuracy of 86% was achieved using SVM with the top 3 input features. (C) Performance using SVM and top 3 features. Left: AUC of 0.92 was achieved. Right: The confusion matrix with a sensitivity of 89.8% and a specificity of 81.1%. AI, arousal index; DT, Decision Tree; HB, hypoxic burden; KNN, K‐Nearest Neighbors; NN, Neural Network; ODI, oxygen desaturation index; RF, Random Forest; SVM, Support Vector Machine.

According to the NCA method, the baseline features were ranked in the following order of importance in distinguishing between the two groups: AI, BAI, total HB, apnea index, AHI, apnea‐specific HB, sex, age, ODI, hypopnea‐specific HB, and hypopnea index (Fig. 5A).

We employed SVM, RF, DT, KNN, and NN models to develop CPAP effectiveness classifiers, using eight feature set combinations (Fig. 5): all features, top 1, 3, and 5 features by feature importance ranking, EEG‐based features only, features excluding EEG measures, features without BAI, and BAI‐only features (Fig. 5A). SVM with a cubic polynomial kernel and top 3 features showed the highest accuracy at 86%, followed closely by SVM with top 5 features, SVM with all features, RF with top 3 features, and RF with top 5 features (Fig. 5B). The peak accuracy model, SVM with top 3 features, achieved an AUC of 0.92, sensitivity at 89.8%, and specificity at 81.1% (Fig. 5C).

For a home sleep study scenario (level 3) where EEG is not used, the RF model achieved 81% accuracy without EEG features that included AI and BAI. On the other hand, when using only EEG‐based features, SVM and DT models achieved the highest accuracy of 83%. When using BAI only, NN and SVM achieved 79% accuracy. Using all baseline features except BAI, SVM and RF reached the highest accuracy of 83%.

## Discussion

Our study pioneers the assessment of CPAP effectiveness on brain health using sleep EEG‐based BAI. Our findings provide compelling evidence that CPAP treatment leads to a significant reduction in the brain age index among OSA patients compared to those untreated. Notably, this positive impact was especially pronounced in individuals with elevated AI, AHI, HB, and apnea‐related indices at baseline prior to the treatment. Moreover, we successfully demonstrated the feasibility of a predictive model, utilizing machine learning techniques to which baseline clinical features were fed, for forecasting the reduction in brain age index. This predictive tool holds promise for preemptive utilization.

### The effectiveness of CPAP on stopping accelerated brain aging due to OSA

In our previous work, we introduced a sleep EEG‐based brain age prediction model and revealed that individuals with OSA have significantly elevated BAI compared to healthy sleepers.^26^ This increased BAI was linked to cortical thinning in various functional areas, suggesting that OSA‐related elevated BAI might reflect neurophysiological shifts leading to neural decline and altered brain activity.^26^ These changes could potentially be attributed to recurring hypoxia, cerebral hypoperfusion, and sleep fragmentation.^36^

Our current study successfully demonstrates that CPAP treatment holds the potential to partially reverse the pathological brain aging process associated with OSA. Analyzing our SMC dataset, we observed a significant reduction in BAI following CPAP treatment, a trend not observed in the untreated group. Importantly, these findings were replicated when analyzing the ABC dataset, even with a shorter follow‐up treatment period. Notably, these improvements in BAI among CPAP users in both SMC and ABC dataset were observed even with the absence of CPAP use during follow‐up PSG. On the other hand, we found lack of significant changes in sleep parameters at follow‐up PSG. This suggests that the improvements in brain age are attributed to the long‐term changes on the brain, led by persistent CPAP use, independent of its immediate effects on sleep and respiration.

Despite other investigations delving into the impact of CPAP on brain structure and function using neuroimaging or neuropsychiatric tests, our study stands as the first to propose sleep EEG‐based brain age as a novel biomarker for assessing the efficacy of CPAP treatment on brain health. This approach holds practical benefits as PSG is a standard diagnostic procedure for OSA in sleep clinics, rendering it a more pragmatic tool than neuroimaging or neuropsychiatric assessments.

### Baseline patient characteristics as predictors of CPAP's therapeutic effect on brain aging

Although we did observe a significant reduction in BAI over time within the CPAP‐treated group, it is noteworthy that the standard deviation of BAI increased from 6.4 to 7.4 years after treatment in comparison to the baseline (Fig. 1). This heightened variance indicates varying responses to CPAP treatment among OSA patients. Some experience distinct brain health improvements, while others don't respond as positively. This diversity in outcomes might shed light on mixed results in prior studies probing CPAP's impact on brain structure and function. Hence, our study underscores pretreatment assessment's importance to identify potential CPAP responders, impacting treatment decisions and shaping future research.

In the analysis exploring associations between the change in BAI following CPAP treatment and baseline characteristics, we found that a decrease in BAI was associated to higher BAI, AI, AHI, HB, apnea index, and older age. Notably, higher AHI and, HB (total and apnea‐specific) were also significantly correlated with an increase in BAI over time in untreated individuals. These findings suggest that individuals with more severe OSA could potentially benefit more from CPAP treatment in terms of mitigating brain aging. Consistent with these findings, as shown in Figure 4B, the good responders (who showed a decrease in BAI) had baseline scalograms that displayed lower power of slow wave components compared to poor responders.

A noticeable observation is that the baseline AI exhibited the most pronounced association among PSG indices with a decrease in BAI following CPAP, implying that consistent CPAP usage might considerably enhance sleep continuity and subsequently reverse the pathologic brain aging seen in OSA (Fig. 3B). Microarousals stemming from disrupted sleep are known to increase oxidative stress due to the activation of stress pathways in the brain^37^, ^38^ and potentially hinder the glymphatic clearance system^39^, thereby accelerating brain aging through the accumulation of waste metabolites like amyloid beta protein.

Another notable finding is that apnea‐related indices, namely the apnea index and apnea‐specific HB, exhibited a significant correlation with the reduction of BAI. In contrast, hypopnea‐related indices, including the hypopnea index and hypopnea‐specific HB, did not demonstrate a significant relationship and showed weak correlation, respectively. One could speculate that the impact of pathologic brain aging is influenced more profoundly by a single severe respiratory event as opposed to multiple minor respiratory events. However, further research is needed to fully understand the specific interactions between respiratory events, brain health, and the therapeutic effects of CPAP treatment as the underlying mechanism remains unclear.

Among the nonrespiratory measurements, high baseline BAI was significantly associated with decrease in BAI following CPAP. This could be interpreted as suggesting that brains more severely impacted by OSA, either due to the presence of more severe OSA or more arousals, or greater susceptibility of the brain, might experience more substantial improvement with CPAP treatment. Interestingly, indices associated with CPAP adherence did not demonstrate a significant correlation with changes in BAI. This absence of correlation might be attributed to the traits of the participants in the CPAP group, who consistently maintained CPAP therapy and typically showed good adherence. These findings suggest that, beyond a certain level of adherence, the relationship between adherence and therapeutic effect doesn't follow a straightforward dose–response pattern.

### The utility of prediction model for CPAP effectiveness

Predicting CPAP effectiveness prior to treatment initiation offers multifaceted benefits. It not only aids in selecting the most suitable patients for treatment but also acts as a motivating factor, boosting patient commitment even before the treatment starts. While recent endeavors have focused on devising prediction models for CPAP adherence, a model specifically for CPAP effectiveness is lacking in current literature.^40^, ^41^ This gap may arise from the lack of suitable outcome metrics to assess the effectiveness of CPAP. In our study, we utilized the reduction in ΔBAI as a relevant outcome measure to develop a prediction model for evaluating the impact of CPAP on brain health. Our developed model, incorporating AI, BAI, and HB, exhibited an impressive accuracy of 86% and an AUC of 0.92. Additionally, our model achieved a slightly lower accuracy of 81%, even when relying solely on non‐EEG‐based parameters like HB, apnea index, AHI, age, sex, ODI, and hypopnea index for CPAP effectiveness prediction. This implies that our model can be effectively extended to patients undergoing portable respiratory monitoring, a widely embraced approach for simplified OSA diagnosis in contemporary clinical settings.

However, our model's identification of poor CPAP responders doesn't negate treatment need. CPAP decisions shouldn't solely consider brain age but also broader impacts like cardiovascular health, metabolism, and quality of life.^42^, ^43^, ^44^, ^45^ Thus, while guided by our model, treatment choices must embrace diverse outcomes, extending beyond brain aging, for a comprehensive effectiveness prediction framework.

### Limitations

This study acknowledges several limitations. Firstly, while we focused on changes in sleep EEG‐based brain age, we didn't examine its relation to changes in brain structure or cognitive function due to the lack of pre‐ and post‐CPAP imaging or neuropsychological data. However, it is pertinent to note that prior studies have documented CPAP's influence on brain structure^15^, ^17^, ^46^ and cognitive function.^18^, ^19^, ^20^ Furthermore, existing evidence links increased EEG‐derived BAI with cortical thinning.^26^ Future studies could delve deeper into these associations. Secondly, when comparing the CPAP‐treated and untreated groups, differences in age and severity of OSA were evident. These variations potentially signify distinct phenotypes between the groups and might introduce inherent bias into the outcomes, complicating direct interpretations. Importantly, this divergence in OSA severity underscores recruitment challenges, particularly concerning untreated patients who might have encountered insufficient treatment effects during the initial 2 weeks of CPAP initiation (potentially explaining the lower AHI and HB in the untreated group). Consequently, a higher proportion of patients with milder OSA could be included in the untreated group.^47^ To mitigate potential biases and enhance interpretability, we diligently adjusted for all conceivable confounders by incorporating them as covariates in linear regression analyses while comparing the two groups. Third, the time intervals in the SMC dataset varied widely, from 1 to 9 years, which could obscure the exact impact of CPAP. To address this concern, we adopted both ΔBAI and annual ΔBAI as outcomes. Fortunately, we managed to replicate our SMC dataset findings using the ABC dataset, where the follow‐up period was standardized at 18 months. Fourthly, the limited sample size (*N* = 98) for our CPAP effectiveness prediction model might reduce statistical power, affecting model performance and increasing overfitting risk. Despite applying five‐fold cross‐validation by partitioning the limited dataset into subsets, the model's efficacy could be compromised. As highlighted in the third limitation, future studies with larger samples could enhance model performance and validate our initial findings. Lastly, the low proportion of females in the study groups (9.2% in CPAP treated and 19.3% in untreated) may have limited our ability to produce entirely unbiased gender‐related outcomes, despite our efforts to adjust for sex‐related confounding effects in the analysis. This underscores the necessity for gender‐balanced recruitment in future studies, to ensure more equitable and comprehensive findings.

## Conclusion

The outcomes of this study provide substantial support for the efficacy of CPAP treatment in counteracting pathologic brain aging among patients with OSA. Notably, this beneficial impact appears more pronounced in patients exhibiting severe OSA, characterized by elevated apnea index, HB, and AHI. Additionally, individuals with fragmented sleep and advanced brain age likely derive greater advantages from a long‐term CPAP use concerning brain aging. Sleep EEG‐derived BAI holds significant potential as a biomarker to gauge the impact of CPAP on brain aging, and its integration into a machine learning‐driven predictive model for CPAP effectiveness is promising. Its wide availability, as it can be conveniently derived from sleep EEG data, commonly collected during standard polysomnography, is likely to propel its increasing utilization in the diagnosis and treatment of OSA.

## Author Contributions

SY: Formal analysis, methodology, validation, and writing—original draft. HRP: Conceptualization, data curation, validation, and writing. EYJ: Project administration, supervision, writing—review and editing, and funding acquisition. HK: Supervision, conceptualization, and writing—review and editing.

## Conflict of Interest

This research was supported by a grant from the PHILIPS Sleep & Respiratory Care (S&RC, PHO0223151).

## Supporting information


Figure S1.

## Data Availability

Source code for the proposed brain age prediction model is available (https://github.com/shyook83/EEG‐BAI). Derived data supporting the findings of this study are available from the corresponding author upon reasonable request.
