# Supplementary material for: Predicting the impact of CPAP on brain health: A study using the sleep EEG‐derived brain age index
Source: Ann Clin Transl Neurol. 2024 Feb 23;11(5):1172–83. doi: 10.1002/acn3.52032 (PMC11093235; doi:10.1002/acn3.52032)
Supplement: Supplementary file 1 — Figure S1. [file ACN3-11-1172-s001.docx]

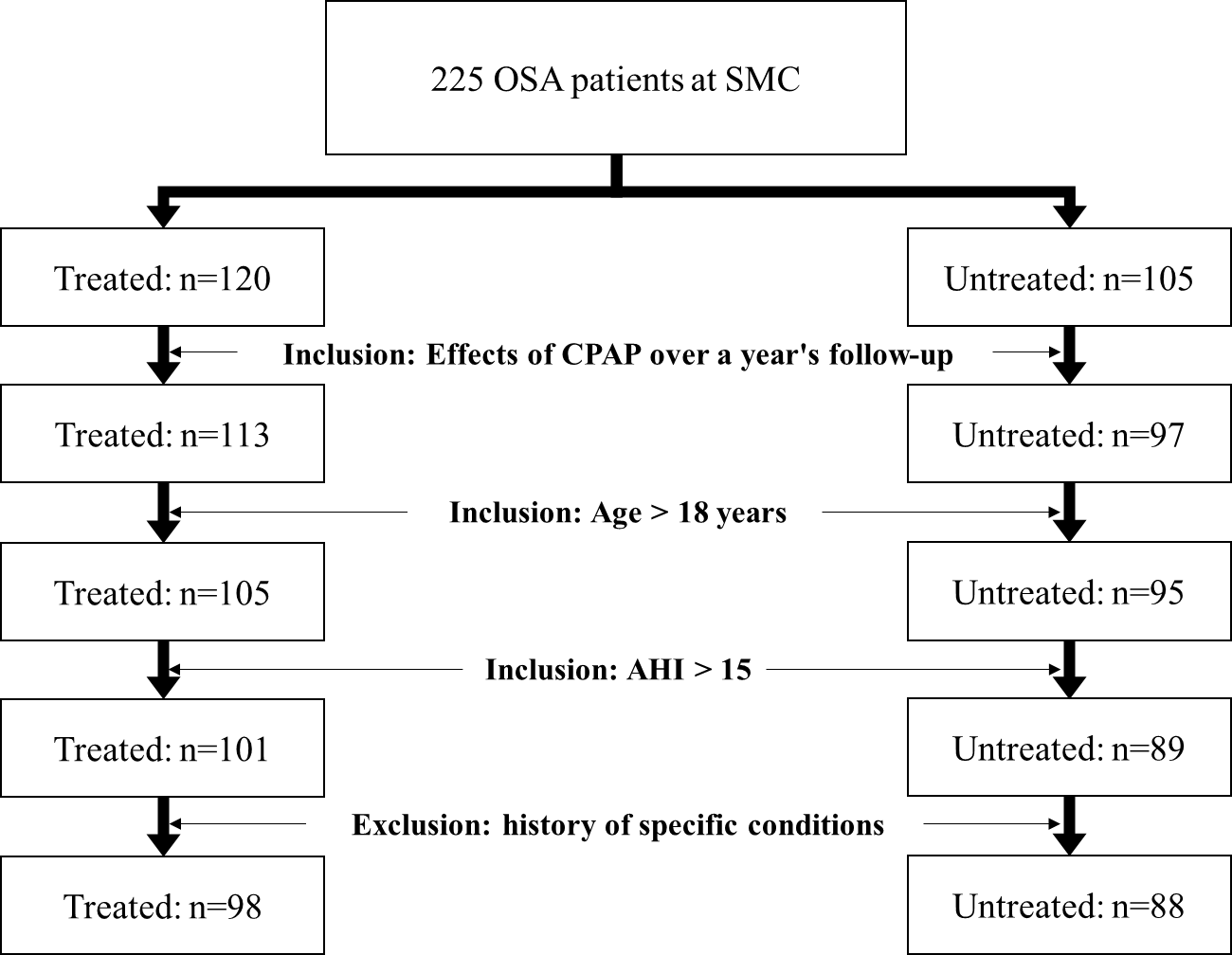


**Figure S1.** This flow diagram outlines the inclusion and exclusion process for a study on Obstructive Sleep Apnea (OSA) patients at Samsung Medical Center. Starting with an initial pool of 225 patients, the diagram bifurcates into two treatment statuses: 'Treated' and 'Untreated,' with 120 and 105 patients, respectively. For long-term effects of Continuous Positive Airway Pressure (CPAP), only those with over a year's follow-up are considered, narrowing the numbers to 113 treated and 97 untreated patients. Further inclusion criteria, such as being above 18 years, having an Apnea-Hypopnea Index (AHI) greater than 15, and no history of cerebrovascular, neurological, or psychiatric conditions, are applied. This results in 98 treated patients (10 of whom are female) and 88 untreated patients (21 of whom are female) for the final analysis. The follow-up duration for the treated group is 4.7±2.5 years, and for the untreated group, it is 4.2±2.4 years.


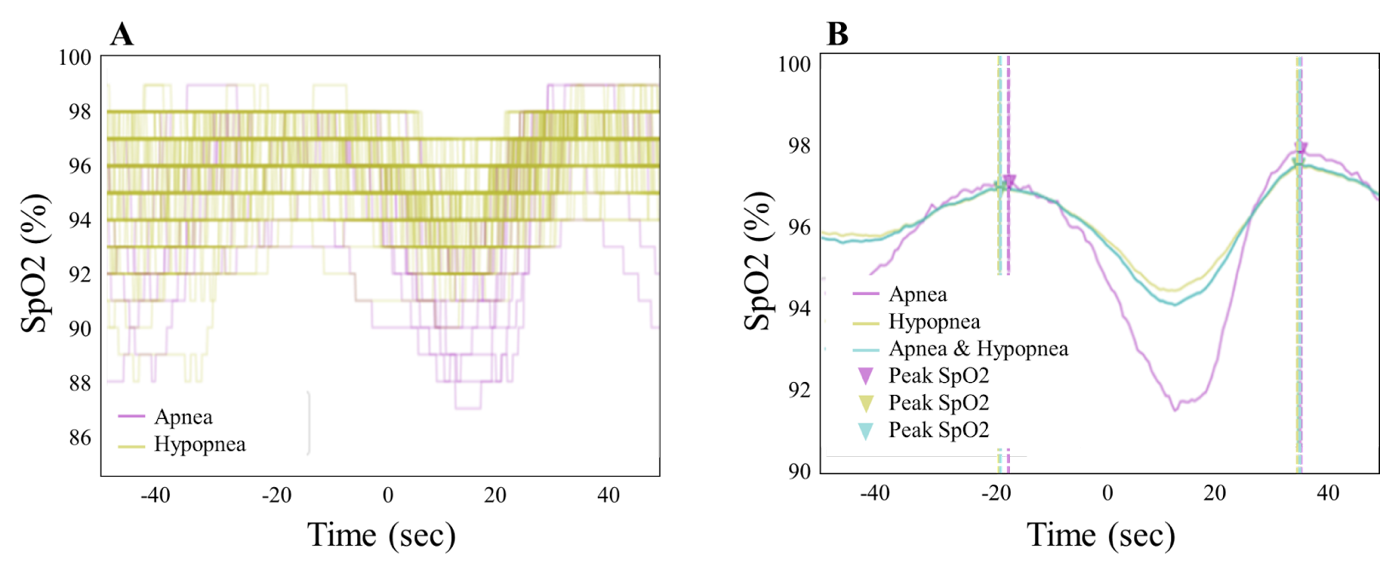
 **Figure S2.** For hypoxic burden (HB) calculation, we adopted the method suggested by Ali Azarbarzin^2^ and developed its concept by additionally analyzing respiratory event-specific HB. Initially, we identified the SpO2 period corresponding to the manually annotated respiratory event for HB calculation, consistent with the conventional method. In each subject, we designated the end point of the respiratory event as time point zero in the synchronized SpO2 signal. From these zero points, we extracted a total duration of 100 seconds, comprising 50 seconds before and 50 seconds after. When all these extracted segments were overlapped in the single subject, the result resembled Supplementary Figure 3A. We then averaged the overlapped SpO2 curves and identified the two highest SpO2 values as peaks, as represented by the blue triangles in Supplementary Figure 3B.

Next, we set the interval between these peaks as the 'search window' and applied this window to all apnea/hypopnea event segments in the SpO2 signal, based on the aforementioned time point zero. Within this search window, the maximum SpO2 was defined as the baseline, and the area between this baseline SpO2 and the SpO2 curve was calculated for each respiratory event. Finally, the Total HB was determined by dividing the sum of these areas by the total sleep time.

This original method of deriving the average SpO2 curve, search window, and HB was applied irrespective of the types of respiratory events and whether a desaturation of more than 3% or 4% occurred. For a more detailed analysis, we defined the apnea-specific HB as the HB calculated solely from apnea events, and the hypopnea-specific HB as the HB calculated only from hypopnea events. When calculating the apnea-specific HB, we identified the peak points of the search window considering only apnea events, as indicated by the magenta triangles in Supplementary Figure 3A. Conversely, for the hypopnea-specific HB, we defined the peak points considering only hypopnea events, as shown by the yellow triangles in Supplementary Figure 3A.

**
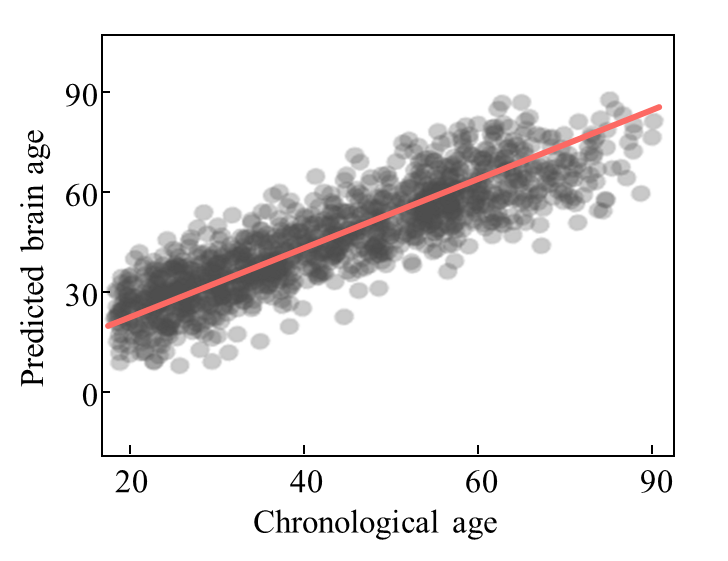
**

**Figure S3.** In comparison to chronological age, our brain age prediction model that estimated ages for healthy subjects (n=1,259) from Samsung medical center attained a Pearson's correlation coefficient of r=0.86 and a mean absolute error (MAE) of 4.8 years.^1^

**Table S1.** Comparison of Baseline and Follow-Up Sleep Parameters for CPAP-Treated and Untreated Groups. No differences were observed between baseline and follow-up in CPAP-treated and untreated groups. Furthermore, changes in sleep parameters from baseline to follow-up (ΔX) showed no correlation with changes in BAI (ΔBAI).

|  | **CPAP-treated** | | | | | **Untreated** | | | | | |
| --- | --- | --- | --- | --- | --- | --- | --- | --- | --- | --- | --- |
|  | Baseline | Follow-up | Baseline vs. Follow-up | Correlation between ΔX and ΔBAI | | Baseline | Follow-up | Baseline vs. Follow-up | Correlation between ΔX and ΔBAI | |  |
|  |  |  | *p* | *r* | *p* |  |  | *p* | *r* | *p* |  |
| Epworth sleepiness score | 10.4 (4.9) | 9.6 (4.9) | 0.09 | 0.01 | 0.91 | 9.7 (5.3) | 9.5 (5.0) | 0.35 | 0.12 | 0.41 |  |
| Total sleep time, min | 363.9 (57.0) | 350.5 (84.0) | 0.31 | 0.07 | 0.49 | 370.0 (58.1) | 370.1(83.6) | 0.37 | 0.07 | 0.63 |  |
| Sleep latency, min | 9.0 (16.1) | 7.3 (12.6) | 0.82 | 0.04 | 0.68 | 10.6 (22.5) | 22.1 (66.5) | 0.35 | 0.10 | 0.49 |  |
| WASO, min | 64.2 (36.9) | 56.0 (47.2) | 0.37 | 0.08 | 0.43 | 62.3 (52.2) | 52.0 (35.9) | 0.15 | -0.22 | 0.11 |  |
| Sleep efficiency, % | 83.4 (8.7) | 85.4 (10.3) | 0.60 | -0.08 | 0.44 | 83.8 (12.6) | 83.6 (16.8) | 0.44 | 0.18 | 0.18 |  |
| N1, % | 29.1 (13.8) | 25.3 (12.6) | 0.71 | -0.05 | 0.63 | 22.1 (12.8) | 18.1 (6.9) | 0.02 | -0.01 | 0.94 |  |
| N2, % | 49.0 (12.7) | 54.8 (11.6) | 0.28 | -0.05 | 0.63 | 54.5 (11.4) | 58.9 (9.4) | 0.24 | 0.05 | 0.74 |  |
| N3, % | 2.4 (3.6) | 2.1 (3.9) | 0.71 | 0.02 | 0.86 | 3.4 (4.9) | 1.8 (3.6) | 0.32 | 0.03 | 0.84 |  |
| REM, % | 19.6 (6.4) | 17.8 (5.4) | 0.09 | 0.15 | 0.15 | 20.0 (6.9) | 21.2 (6.9) | 0.11 | -0.07 | 0.61 |  |
| Apnea–hypopnea index, /h | 42.1 (21.7) | 43.0 (21.6) | 0.67 | -0.02 | 0.84 | 23.4 (16.6) | 25.8 (21.5) | 0.23 | 0.19 | 0.17 |  |
| Arousal index, /h | 37.7 (17.4) | 31.9 (13.4) | 0.25 | -0.003 | 0.97 | 30.1 (36.4) | 24.7 (13.1) | 0.33 | -0.02 | 0.86 |  |
| Total hypoxic burden | 151.2 (160.3) | 133.3 (108.5) | 0.48 | -0.03 | 0.75 | 62.3 (75.5) | 68.2 (85.6) | 0.16 | 0.27 | 0.03 |  |

X: The corresponding sleep parameter

ΔX: Follow-up – baseline

No significant after Bonferroni correction (p<0.05/12)

**Table S2. Baseline demographics and clinical characteristics of ABC dataset**

|  | **CPAP-ABC (n=13)** |
| --- | --- |
| Age | 48.7 (10.3) |
| Men, No. (%) | 7 (53.8) |
| Race | White: 10 / African: 1 / Unknown: 2 |
| Body mass index, kg/m^2^ | 38.5 (3.3) |
| Epworth sleepiness score | 10.2 (5.2) |
| Total sleep time, min | 388.7 (78.0) |
| Sleep efficiency, % | 83.4 (8.7) |
| N1, % | 23.0 (14.2) |
| N2, % | 48.2 (9.0) |
| N3, % | 10.7 (9.0) |
| REM, % | 18.1 (6.9) |
| Apnea–hypopnea index, /h | 43.1 (31.2) |

**S1:** Methodology for Brain Age Index (BAI) prediction

1. Data Collection and Initial Setup:

Sleep EEG data was sourced from eight distinct channels: F3, F4, C3, C4, O1, O2, A1, and A2 along with sleep stage. A1 and A2 served as reference channels. A total of 1,259 healthy sleepers' data was utilized to construct the brain age prediction model.

2. EEG Preprocessing and Artifact Removal:

EEG readings were captured from six distinct channels: frontal, central, and occipital, at a sampling rate of 200 Hz. The data underwent a filtering process within a 0–50 Hz range to eliminate unwanted frequencies and to cleanse it of ECG and EOG-related disturbances. Signal spikes exceeding 5 standard deviations in power were flagged as anomalies and underwent interpolation for correction. In instances where a channel displayed artifacts surpassing 30%, it was substituted with data from its counterpart in the opposite hemisphere. Subsequently, the EEG readings were standardized using the z-score method.

3. Transformation of EEG to Scalogram:

The refined EEG data was transformed into scalograms using the continuous wavelet transform, resulting in six images, each with dimensions of 2000 × 16 pixels. Sleep stage annotations, based on the hypnogram, were also converted into a 2000 × 16 pixels image format. This sleep stage image was then concatenated with the six scalogram images, culminating in an input data structure of 2000 × 16 × 7 dimensions.

4. Development of the Brain Age Estimation Model:

The model foundation was built on the DenseNet framework, a variant of the Convolutional Neural Network (CNN). The Mean Absolute Error (MAE) between the actual chronological age and the model's predicted age served as the performance metric and loss function.

5. Derivation of the EEG-Based Brain Age Index (BAI):

The BAI is derived by taking the difference between the chronological age and the age predicted by the model. To ensure the accuracy of the BAI, adjustments were made to counteract any inherent biases in the regression models, thus providing a more precise gauge of an individual's relative neurological health.

.

References

1. Yook S, Park HR, Park C, et al. Novel neuroelectrophysiological age index associated with imaging features of brain aging and sleep disorders. NeuroImage. 2022;264:119753.

2. Azarbarzin A, Sands SA, Stone KL, et al. The hypoxic burden of sleep apnoea predicts cardiovascular disease-related mortality: the Osteoporotic Fractures in Men Study and the Sleep Heart Health Study. European heart journal. 2019;40(14):1149-57.
